# Myelin oligodendrocyte glycoprotein antibody-associated isolated seizures in children: a retrospective single-center case series

**DOI:** 10.3389/fimmu.2026.1934764

**Published:** 2026-10-07

**Authors:** Kang Liu, Panhui Yu, Liyao Ma, Yubing Gong, Hongru Lu, Wei Wang, Fang Chen

**Affiliations:** 1 First Department of Neurology, Hebei Children’s Hospital, Shijiazhuang, China; 2 Hebei Clinical Medicine Research Center for Children’s Health and Diseases, Shijiazhuang, China; 3 Hebei Provincial Key Laboratory for Pediatric Epilepsy and Neurological Disorders, Shijiazhuang, China

**Keywords:** children, focal motor seizure, isolated seizure, myelin oligodendrocyte glycoprotein antibody, unremarkable cranial MRI

## Abstract

**Background and purpose:**

Isolated seizures represent a distinct, rare, and underrecognized phenotype of myelin oligodendrocyte glycoprotein (MOG) antibody-associated disease (MOGAD). In recent years, documentation of this phenotype has been limited to sporadic reports, with dedicated case series being extremely scarce. This study aimed to describe the clinical characteristics of isolated seizures in pediatric patients with MOGAD.

**Methods:**

We retrospectively reviewed the medical records of pediatric patients diagnosed with MOG antibody-associated isolated seizures between January 2019 and January 2026. Clinical manifestations, brain magnetic resonance imaging (MRI) findings, laboratory data, treatment regimens, and clinical outcomes were systematically analyzed.

**Results:**

Among the cohort of 172 pediatric patients with seropositive MOG antibodies, 6 (3.4%) met the inclusion criteria for isolated seizures. The median age at onset was 7.5 years (range, 5–9 years), with a male-to-female ratio of 1:2. Clinically, 4 patients presented solely with epileptic seizures, while the other 2 patients had additional transient limb weakness or headache, respectively. Focal seizures were observed in 5 patients, including 3 with focal motor seizures; additionally, 2 cases experienced status epilepticus. No patients exhibited encephalopathy or focal neurological deficits. Four patients had peripheral blood leukocytosis (range, 11.9–26 × 10^9^/L), and five had elevated erythrocyte sedimentation rates (ESR; range, 22–43 mm/h). All six patients showed cerebrospinal fluid (CSF) pleocytosis (41.1 ± 20.4/μL; range, 15–69/μL) without detectable infectious pathogens. Abnormal electroencephalogram (EEG) findings, including abnormal background activity and focal slow waves, were identified in 3 patients. No cranial MRI abnormalities were detected in any patient. All patients received immunotherapy, with one patient additionally receiving anti-seizure medications (ASMs). At final follow-up, all patients had favorable outcomes, with no recurrence of seizures or subsequent demyelinating events.

**Conclusion:**

Myelin oligodendrocyte glycoprotein antibody-associated isolated seizures is a rare and frequently underrecognized phenotype within the spectrum of MOGAD. For school-age children presenting with epileptic seizures, particularly focal motor seizures, along with elevated ESR and CSF pleocytosis, early testing for MOG antibody is recommended in the absence of clinical or radiological evidence of encephalitis or focal neurological deficits. Most patients achieve favorable clinical outcomes, which may be partially attributed to immunotherapy. However, the efficacy of immunotherapy in preventing seizure recurrence, subsequent demyelinating events, and subclinical radiological disease remains indeterminate.

## Introduction

Myelin Oligodendrocyte Glycoprotein (MOG) is an N-type, membrane-bound glycoprotein uniquely expressed on the membranes of oligodendrocytes within the central nervous system (CNS). Predominantly located in the outer layer of myelin lamellae, MOG accounts for about 0.05% of CNS myelin proteins and is essential for maintaining myelin integrity and stability (1). Due to its high immunogenicity, MOG may act as a key target for autoimmune antibodies and cellular immune responses in inflammatory demyelinating CNS diseases (2). Cell-based assays (CBAs) facilitate the expression of MOG epitopes with the proper three-dimensional structure on cell surfaces, thereby allowing for selective binding to pathogenic antibodies (3). Thus, CBAs have become the most widely used method for detecting MOG antibodies.

Myelin oligodendrocyte glycoprotein antibody-associated disease (MOGAD) is currently acknowledged as a distinct disease entity, differing from multiple sclerosis (MS) and aquaporin-4 antibody-positive neuromyelitis optica spectrum disorder (AQP4-positive NMOSD) in both clinical and immunopathological profiles. The prevalence of MOGAD is approximately 1.3–2.5 per 100,000, with an annual incidence of 3.4–4.8 per 100,000, and this disease occurs more frequently in pediatric populations than in adults (4). In recent years, the relationship between epileptic seizures and MOG antibodies has attracted considerable academic interest, as seizures are far more common in MOGAD (20.5%) than in MS (2%) and AQP4-positive NMOSD (0.35–0.7%) (5, 6). Epileptic seizures may present across various clinical phenotypes of MOGAD, with the highest incidence reported in cerebral cortical encephalitis (CCE) and acute disseminated encephalomyelitis (ADEM)-like phenotypes (5, 7, 8). Notably, epileptic seizures may occur either concomitantly with or prior to demyelinating events, constituting a clinical feature specific to MOGAD. Accordingly, patients who experience seizures that occur chronologically distinct from any demyelinating event and exhibit unremarkable brain MRI findings are classified as having “isolated seizures”, a rare and often overlooked clinical phenotype in MOGAD.

To date, research on MOG antibody-associated isolated seizures remains limited, particularly concerning the pediatric population, leaving the clinical characteristics and prognosis poorly understood. In this study, through reporting six pediatric patients with MOG antibody-associated isolated seizures, we delineate the clinical features, thereby supporting earlier diagnosis and more effective clinical management of this rare and frequently underrecognized condition.

## Patients and methods

This retrospective study reviewed the clinical data of pediatric patients admitted to Hebei Children’s Hospital between January 2019 and January 2026. All eligible participants met the following inclusion criteria: (i) presentation with the first acute episode of MOGAD; (ii) age ≤ 168 months (14 years); (iii) epileptic seizures as the sole presenting symptom, with unremarkable brain magnetic resonance imaging (MRI) and no concomitant clinical demyelinating events; (iv) positive serum myelin oligodendrocyte glycoprotein immunoglobulin G (MOG-IgG), negative aquaporin-4 immunoglobulin G (AQP4-IgG), and negative testing for other autoimmune antibodies in both serum and cerebrospinal fluid (CSF), including NMDAR, AMPA1, AMPA2, GABAB, LGI1, CASPR2, GAD65, mGluR5, GFAP, as well as Hu, Yo, Ri, Ma2, CV2, Amphiphysin, ANNA-3, Tr, PCA-2, and GAD antibodies. The exclusion criteria were as follows: (i) fulfillment of encephalitis diagnostic criteria established by the International Encephalitis Consortium (9); (ii) a prior history of encephalitis or central nervous system inflammatory demyelinating diseases (CIDDs) within 3 months before the onset of isolated seizures; (iii) patients exhibiting acute-phase disease progression who were ultimately diagnosed with encephalitis or CIDDs; and (iv) seizures due to other causes, including systemic disorders, genetic metabolic diseases, or other autoimmune or inflammatory conditions. Seizure classification was performed in accordance with the 2017 International League Against Epilepsy (ILAE) criteria (10).

We systematically collected and analyzed comprehensive clinical data, including epidemiology (age, gender, medical history), clinical manifestations, laboratory results, electroencephalogram (EEG) and brain magnetic resonance imaging (MRI) findings, treatment regimens, and clinical outcomes. Serum MOG antibodies were detected using either live cell-based assays (live CBAs) or fixed cell-based assays (fixed CBAs), whereas CSF MOG antibodies were exclusively detected via fixed CBAs. All assays were conducted in full accordance with standardized operating procedures (SOPs). Based on the laboratory’s validated cut-off values, MOG antibody titers were categorized as follows: high-positive (≥ 1:100), low-positive (1:10 to 1:32), and negative (< 1:10). Following hospital discharge, all patients received regular outpatient follow-up.

The study received approval from the Medical Ethics Committee of Hebei Children’s Hospital, Hebei Medical University. Written informed consent was obtained from the parents or legal guardians of the participating pediatric patients.

## Results

### Demographics and clinical features

Of the 172 pediatric patients with positive serum MOG-IgG screened throughout the study period, 6 (6/172, 3.4%) met the inclusion criteria. The median onset age was 7.5 years (IQR, 5–9 years), with a male-to-female ratio of 1:2. None of these six patients had a prior history of CNS disease or autoimmune disease. Demographic and clinical characteristics of the participants are detailed in **Table 1**.

**Table 1:** Detailed demographic and clinical characteristics of 6 patients with MOG antibody-associated isolated seizures.

Variables	Patient 1	Patient 2	Patient 3	Patient 4	Patient 5	Patient 6
Demographics						
Age (year)	5	6	9	9	9	5
Sex	F	M	F	F	F	M
**History**	**━**	━	━	━	━	━
Clinical presentation						
Seizure type	FS	FS	FS	FS/ FBTCS	FS	FBTCS
Cluster seizures	+	━	━	━	━	━
Status epilepticus	━	+	━	━	━	+
Headache	━	━	━	━	━	+
Transient limb weakness	━	+	━	━	━	━
Laboratory findings						
Blood						
WBC (10^9^/L)	6.6	**26**	9.7	**11.9**	**20**	**13.8**
CRP (mg/L)	**42**	8.7	6.1	0.6	<0.5	**15.3**
ESR (mm/h)	**43**	**22**	**26**	**30**	ND	**23**
PCT (μg/L)	<0.04	**0.09**	<0.04	ND	ND	**0.11**
CSF						
ICP (mmH_2_O)	ND	ND	180	150	ND	ND
WBC (cells/μL)	**15**	**60**	**25**	**69**	**38**	**40**
Protein (g/L)	0.27	0.29	0.27	0.18	0.17	0.22
Glucose (mmol/L)	3.19	3.28	2.68	2.94	3.11	3.12
Antibodies						
Serum MOG-IgG	**1:1000**	**1:10**	**1:32**	**1:100**	**1:32**	**1:100**
CSF MOG-IgG	━	━	━	━	━	1:10
CSF-specific OCBs	━	ND	━	ND	━	ND
EEG findings						
Abnormal background activity	+	+	━	━	━	━
Focal slow waves	+	+	+	━	━	━
Treatment						
Latency (day)	1	6	24	14	8	8
Anti-infective drug	+	+	+	+	+	+
Immunotherapy	IVMP IVIG	IVMP IVIG	IVMP IVIG	IVMP IVIG	IVMP IVIG	IIVMP IVIG
ASM	PER	━	━	━	━	━
**Length of stay (day)**	23	19	24	32	20	28
Outcome						
Recovery	+	+	+	+	+	+
Follow-up						
Duration (month)	9	34	60	72	62	57
Relapse	━	━	━	━	━	━

ASM, anti-seizure medication; CRP, C-reactive protein; CSF, cerebrospinal fluid; EEG, electroencephalogram; ESR, erythrocyte sedimentation rate; F, female; FBTCS, focal to bilateral tonic-clonic seizure; FS, focal seizure; ICP, intracranial pressure; IVIG, intravenous immunoglobulin; IVMP, intravenous methylprednisolone; M, male; MOG, myelin oligodendrocyte glycoprotein; MOG-IgG, myelin oligodendrocyte glycoprotein immunoglobulin G; ND, no data available; OCBs, oligoclonal bands; PCT, procalcitonin; PER, perampanel; WBC, white blood cell. Values in bold indicate abnormal results (elevated values or MOG antibody titers).

Five patients presented with epileptic seizures as the initial symptom, while one patient initially complained of headache. Focal seizures were observed in 5 patients, and 2 patients had focal-to-bilateral tonic-clonic seizures. In addition, two cases experienced status epilepticus, including one (Patient 2) with transient unilateral limb weakness. Regarding seizure frequency, one case (Patient 1) presented with cluster seizures and showed a poor response to sedative therapy. Among the other five patients, one had three seizure episodes, another had two episodes, and the remaining three patients each had a single seizure episode.

Notably, none of the enrolled patients exhibited fever, psychiatric or behavioral abnormalities, sleep disturbances, visual deficits, or spinal cord involvement throughout the disease course.

### Laboratory findings

All patients underwent complete blood count (CBC) and C-reactive protein (CRP) testing. Leukocytosis was observed in 4 patients (4/6, 66.7%; range, 11.9–26 × 10^9^/L; reference range, 4–10 × 10^9^/L), and 2 patients exhibited elevated CRP levels (range, 15.3–42 mg/L; reference range, 0–8 mg/L). Procalcitonin (PCT) was assessed in 4 patients, with mild elevation noted in 2 (range, 0.09–0.11 μg/L; reference range, 0–0.06 μg/L). Erythrocyte sedimentation rate (ESR) was measured in 5 patients, all of whom demonstrated elevated values (range, 22–43 mm/h; reference range, 0–10 mm/h).

All six patients exhibited CSF pleocytosis (41.1 ± 20.4/μL; range, 15–69/μL; reference range, 0–10/μL), predominantly consisting of mononuclear leukocytes. Intracranial pressure (ICP) was measured in 2 cases, with normal results. None of the patients had abnormal CSF protein and glucose levels. CSF bacterial cultures and polymerase chain reaction (PCR) assays targeting herpes simplex virus (HSV), Epstein-Barr virus (EBV), cytomegalovirus (CMV), and Mycoplasma pneumoniae (MP) were negative in all patients.

Serum MOG-IgG was positive in all patients, whereas CSF MOG-IgG was negative in all. The titers of serum MOG-IgG ranged from 1:10 to 1:1000. Additionally, CSF-specific oligoclonal bands (OCBs) were performed on 3 patients, all of whom yielded negative results.

Additionally, all six patients had normal immunoglobulin levels and negative serum autoantibodies (including antinuclear antibody, anti-Smith, anti-double-stranded DNA, etc). Four patients underwent blood and urine metabolic screening, with unremarkable findings. In two cases, plasma cytokines, as well as complement components C3 and C4, were within normal ranges. Ferritin and D-dimer levels were normal in one patient (Case 1). No patient experienced recurrent fever, mucocutaneous lesions, arthromyopathic manifestations, or other related clinical signs. Taken together with the laboratory findings, there was no evidence of multi-system or multi-organ involvement, making systemic diseases unlikely.

### Electroencephalogram findings

Electroencephalogram (EEG) was performed for all patients, with 3 cases (3/6, 50%) exhibiting abnormal results, consisting of abnormal background rhythms and focal slow-wave activity. During the monitoring period, one case (Patient 1) had a recorded clinical focal motor seizure with preserved awareness.

### MRI findings

During the acute phase, all six patients underwent initial brain MRI, which demonstrated no cerebral parenchymal lesions (**Figures 1A, E, G**). The median interval from disease onset to initial MRI acquisition was 2 days (range, 2–8 days). Four of the six patients (Patient 1, Patient 4–6) received follow-up brain MRI within 30 days after disease onset. Among these, three scans remained normal, whereas one (Patient 1) exhibited subtle FLAIR hyperintensity involving partial cortex of the right parietal lobe on day 13 post-onset (**Figure 1B**), without concurrent clinical symptoms.

Furthermore, three patients (Patient 1–3) completed brain MRI during the remission phase. For Patient 1, a follow-up brain MRI obtained on day 57 after disease onset demonstrated resolution of the subtle FLAIR hyperintensity in the right parietal lobe (**Figure 1C**). A repeat brain MRI acquired at 4 months post-onset showed no abnormal signals (**Figure 1D**). Patient 2 underwent brain MRI at 33 months post-onset due to headache, which yielded normal findings (**Figure 1F**). For Patient 3, serial follow-up brain MRI scans performed at day 44, 8 months, and 16 months (**Figure 1H**) after disease onset revealed no abnormal findings.

Additionally, orbital and spinal cord MRIs were conducted on all six patients, showing no abnormalities.

### Treatment and outcomes

Detailed treatment and outcomes of the six patients are summarized in **Table 1**. Given the initial suspicion of intracranial infection, all six patients received empirical anti-infective therapy. Considering the potential risks and poor prognosis associated with herpes simplex virus infection, all six patients were administered intravenous acyclovir, and one case additionally received combination treatment with azithromycin.

The median time from disease onset to initiation of immunotherapy was 10.1 days (range, 1–24 days). In this study, all six cases received full-dose intravenous immunoglobulin (IVIG, 1 g/kg/day for 2 days) and high-dose intravenous methylprednisolone (IVMP; 20 mg/kg/day for 3 days, followed by a taper to 10 mg/kg/day for 3 days, a further reduction to 5 mg/kg/day for 3 days, and a final taper to 2.5 mg/kg/day for 3 days before discontinuation), with subsequent transition to oral prednisone (OP, 1.5 mg/kg/day). Due to frequent epileptic seizures, Patient 1 was administered oral perampanel.

All patients achieved clinical remission without any adjunctive immunosuppressive treatment during the acute disease phase. The mean hospital stay was 24.3 ± 4.9 days (range, 19–32 days). All patients exhibited favorable clinical outcomes at discharge.

### Follow-up

After discharge, six patients received oral prednisone with gradual dose tapering over a period of ≥6 months, and no adverse events were observed during the tapering phase. Patient 1 has been maintained on concomitant oral perampanel and prednisone, with ongoing dose tapering and no reported adverse reactions to date.

The mean follow-up duration was 49 ± 23.2 months (range, 9–72 months), during which no relapses or demyelinating events occurred in any patient. At the last follow-up, five patients (except Patient 1) had discontinued oral prednisone, and all children achieved a favorable prognosis with sustained seizure freedom.

## Discussion

In this study, we retrospectively analyzed the clinical features of six pediatric patients with isolated seizures, positive serum MOG-IgG, and no evidence of concomitant encephalitis or demyelinating events. To our knowledge, this is the first case series focusing on Asian children to specifically characterize isolated seizures as a rare, underrecognized phenotype of MOG antibody-associated disease (MOGAD).

A range of cell-surface autoantibodies have been linked to acute symptomatic seizures (ASSs) secondary to autoimmune encephalitis. In particular, LGI1-IgG and CASPR2-IgG may facilitate the progression to chronic epilepsy, thereby necessitating long-term anti-seizure medications (ASMs) (11). Myelin oligodendrocyte glycoprotein (MOG), a surface protein expressed on oligodendrocytes, acts as a critical immune target mediating inflammation and demyelination in the central nervous system (CNS). A recent pediatric study (12) demonstrated that ASSs occur in 36.8% of patients with CNS inflammatory demyelinating diseases (CIDDs). Notably, the incidence of ASS was significantly higher in patients with MOGAD (41.5%) and seronegative CIDDs (43.1%) than in those with anti-aquaporin-4 immunoglobulin G (AQP4-IgG)-positive neuromyelitis optica spectrum disorders (NMOSD) (12.5%) and multiple sclerosis (MS) (13.6%). Moreover, the most common seizure-related phenotype in MOGAD is cerebral cortical encephalitis (CCE), followed by acute disseminated encephalomyelitis (ADEM), indicating that cortical lesions contribute substantially to the development of ASS. Compared with other idiopathic inflammatory demyelinating diseases, CCE and ADEM occur predominantly in MOGAD (13, 14), with a higher incidence in children than in adults (12, 15). Accordingly, age at onset and clinical phenotype may influence the occurrence of ASS. In MOGAD, focal onset seizures (FOSs), particularly focal motor seizures, are the predominant seizure type (16), manifesting either with cortical lesions on MRI or with unremarkable neuroimaging findings. Consistent with previous findings, all six patients in this study showed normal brain MRI results and predominantly exhibited focal seizures (4/6, 66.7%), followed by focal-to-bilateral tonic-clonic seizures (2/6, 33.3%).

In 2018, isolated epileptic seizures without concomitant encephalopathic or demyelinating manifestations were first reported in pediatric patients with MOGAD (17). Subsequent studies have documented this clinical phenotype in patients with leucine-rich glioma-inactivated 1 (LGI1) IgG (18) and contactin-associated protein-like 2 (CASPR2) IgG (19) associated disorders. However, clinical descriptions of MOG IgG-associated isolated seizures remain extremely rare, especially in the pediatric population. In this MOG-IgG-positive cohort, the incidence of the isolated seizures phenotype was 3.4%, exceeding the 1.3% documented in a previous retrospective study (20). This discrepancy is likely attributed to inter-study differences in sample size. Given that isolated seizures at disease onset are difficult to distinguish from encephalitis and other epileptic conditions, coupled with the lack of early detection of MOG-IgG, misdiagnosis and underdiagnosis are common, thereby delaying the prompt recognition of this phenotype. Furthermore, isolated seizures arising following a demyelinating event or encephalitis may be misinterpreted as recurrent episodes of the preceding condition rather than identified as distinct clinical entities. Therefore, increased awareness and further research are warranted to better identify and characterize this rare phenotype.

The median age at onset in this study was 7.5 years, which is comparable to the 6.8 years reported for ASSs in pediatric MOGAD (12), yet slightly lower than the 10.3 years documented in a prior study involving three children with isolated seizures (20). These findings suggest an age-dependent pattern in pediatric MOG-IgG-associated isolated seizures. Among the six pediatric patients, one presented with clustered seizures, three had only a single seizure episode, and two developed status epilepticus, indicating substantial heterogeneity in the frequency and severity of MOG antibody-associated isolated seizures. No other seizure types, including myoclonic seizures, epileptic spasms, absence seizures, or atonic seizures, were identified. A prior study involving six adult patients with MOG antibody positivity and isolated symptomatic seizures (21) found that focal-to-bilateral tonic-clonic seizures were predominant and occurred in clusters. Brain MRI in all cases revealed cortical-subcortical demyelinating lesions, with brainstem and basal ganglia involvement observed in a subset of cases. In contrast, the present study showed a predominance of focal seizures without a cluster pattern, accompanied by unremarkable brain MRI findings. Accordingly, we hypothesize that seizure phenotypes in MOGAD may correlate with structural lesions on brain MRI.

Additionally, clinical findings included nonspecific symptoms (headache and transient limb weakness) in two patients, elevated peripheral white blood cell (WBC) counts in four, mildly elevated CRP and PCT in two patients each, and slow-wave EEG abnormalities in three. Of particular note, all six patients had elevated ESR and CSF pleocytosis with negative infectious etiologies, and all showed substantial clinical improvement following immunotherapy, although clustered seizures in Patient 1 remained refractory to sedatives and ASMs. Due to the lack of specific clinical manifestations and supportive auxiliary examination findings, along with limited relevant research, the primary challenge in managing MOG antibody-associated isolated seizures lies in early recognition and diagnosis. Of note, MOG antibody testing should be recommended for pediatric patients with isolated seizures presenting the following features: school-age onset, focal seizure semiology, no evidence of encephalopathy or demyelination, elevated ESR and CSF pleocytosis, and especially poor response to sedatives and ASMs.

Observations worthy of consideration in this study deserve emphasis. First, Patient 1 presented with cluster seizures and a high MOG-IgG titer (1:1000), whereas Patients 2 and 6 developed status epilepticus from a single seizure episode with low MOG-IgG titers (1:32). Importantly, no patient experienced seizure recurrence during follow-up, suggesting that MOG-IgG titers may not correlate significantly with seizure frequency, severity, or recurrence risk. Second, ASSs in MOGAD may present across a wide range of clinical phenotypes. The relationship between seizures and MOG antibodies remains an important yet unresolved research question, particularly concerning the pathogenesis of seizures in patients with normal cranial MRI. Given that not all MOGAD phenotypes present with seizures, and no significant correlation exists between antibody titers and seizure severity or course, it is hypothesized that MOG antibodies may not directly induce seizures. Potential mechanisms include the following: (1) oligodendrocytes directly adhere to neurons to provide metabolic support (22). MOG antibody-mediated oligodendrocyte injury may impair neuronal stability, thereby triggering seizures; (2) microglia activation and cytokine-driven disruption of ionic homeostasis may increase neuronal excitability (23), with corresponding inflammatory changes not detectable on conventional MRI; (3) synergistic effects with coexisting, yet unidentified autoimmune antibodies analogous to NMDAR antibodies may also be involved.

Isolated seizures represent a rare phenotype lacking distinct clinical features, and relevant research remains limited. As a result, no evidence-based treatment guidelines exist, and controversy persists regarding the efficacy of immunotherapy versus ASMs. In the present study, all six pediatric patients received antiviral therapy as viral encephalitis could not be excluded at disease onset. In accordance with the consensus of the European Pediatric MOG Consortium (24) and the treatment guidelines established by the Chinese Society of Neuroimmunology (25), high-dose IVMP pulse therapy combined with IVIG is recommended in the acute phase, followed by a gradual tapering regimen of IVMP and subsequent administration of oral corticosteroids. The tapering period of oral corticosteroids should be no less than 6 months. Given the potential underlying immunological pathogenesis, all children in this study received the aforementioned immunotherapy regimen, with a median interval of 10.1 days (range, 1–24 days) from disease onset to the immunotherapy initiation. This variability was primarily attributed to differences in the timing of medical care seeking, disease severity, and the duration required to complete diagnostic examinations. Patient 1 received combined ASM due to cluster seizures with poor response to sedative agents. Although there is insufficient data to establish detailed recommendations for managing acute symptomatic seizures, the existing literature suggests that most seizures respond favorably to immunotherapy.

In the first report describing isolated seizures by Ramanathan (17), one patient achieved seizure remission with antiseizure medication (ASM) monotherapy and experienced neither seizure recurrence nor demyelinating events; one patient had spontaneous remission without ASM or immunotherapy; and two patients attained seizure control with ASM. However, the latter three children subsequently developed seizure recurrence and demyelinating events during follow-up. Additionally, some patients with status epilepticus exhibited a poor response to ASMs, further supporting a potential immune-mediated pathogenesis. Subsequent pediatric case reports have also documented seizure recurrence in the absence of immunotherapy (7, 20). In an adult study (26), four patients with isolated epileptic seizures received corticosteroid therapy and remained seizure-free during a follow-up period ranging from 8 to 56 months. These results indicate that immunotherapy may potentially reduce seizure recurrence and demyelinating events in patients with MOG antibody-associated isolated seizures. However, a previous case report described a child with MOG-CCE who relapsed with isolated seizures despite receiving immunotherapy at initial onset, but subsequently achieved sustained seizure freedom with oral ASMs alone (23). Interestingly, inflammatory markers decreased after three months compared with the acute relapse phase, which may be attributed to the anti-inflammatory properties of levetiracetam (LEV) (27) and carbamazepine (CBZ) (28). Consequently, there remains no consensus regarding the acute and maintenance therapeutic regimen for symptomatic seizures in MOGAD. In general, the initiation of ASM in the acute phase necessitates a comprehensive assessment of seizure characteristics, while the long-term use of ASM during the remission phase requires a thorough evaluation of seizure recurrence risk.

In this study, we observed that only one patient (Case 1) developed radiological abnormality on day 13 post-onset, characterized by subtle FLAIR hyperintensity in a portion of the right parietal lobe cortex (**Figure 1B**). This finding was regarded as a cortical lesion resulting from the initial cluster of epileptic seizures. Concurrently, the seizures had been controlled, and no relevant symptoms were present. Importantly, a follow-up brain MRI performed on day 57 after disease onset demonstrated resolution of the abnormal signals. During the remission phase, all patients experienced no clinical relapses. With a mean follow-up duration of 49 ± 23.2 months (range, 9–72 months), all patients in this study experienced no clinical relapses. Three patients (Cases 1–3) completed follow-up brain MRIs without abnormalities, whereas subclinical radiological disease could not be excluded in the remaining three patients. Consequently, the long-term prognosis remains indeterminate in this study.

**Figure 1:**
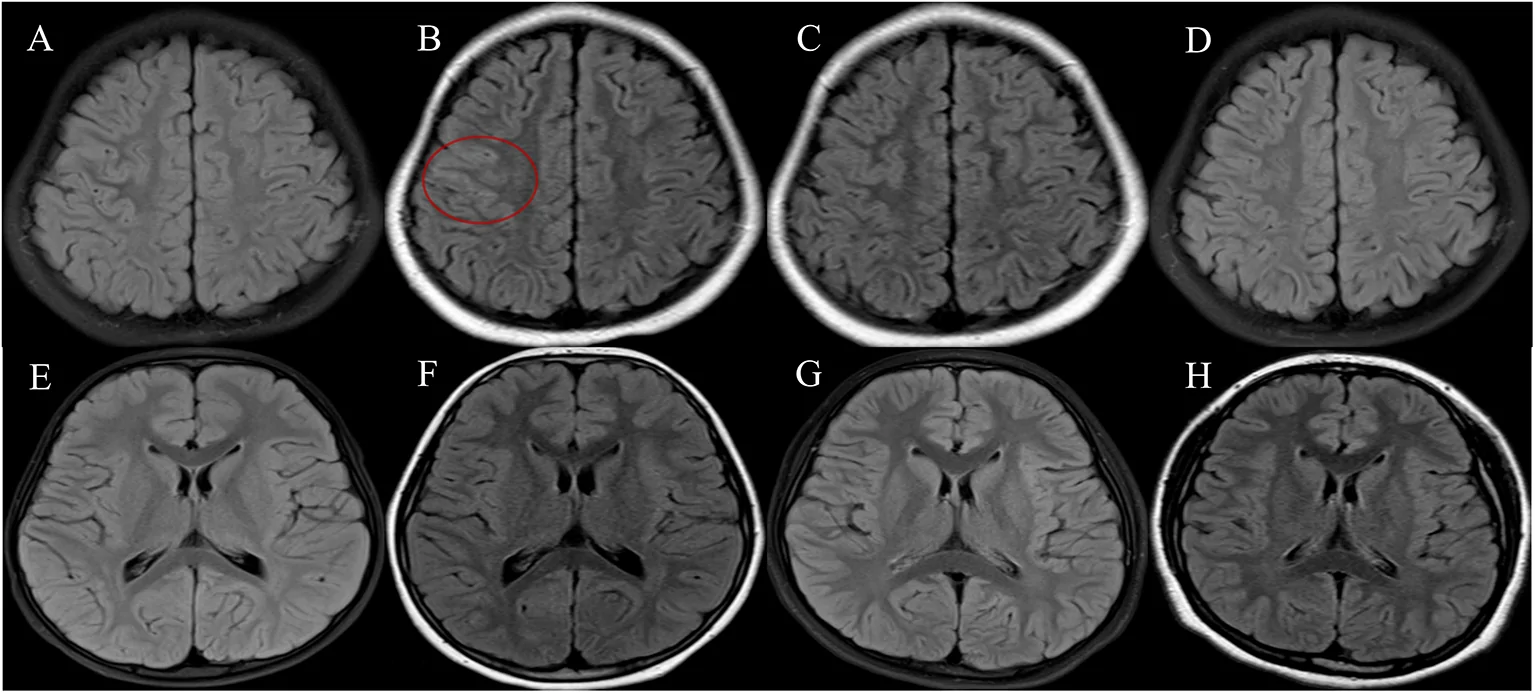
Brain magnetic resonance imaging (MRI) of patients with myelin oligodendrocyte glycoprotein (MOG) antibody-associated isolated seizures. Patient 1: **(A)** Normal brain MRI on day 3 after disease onset; **(B)** Fluid-attenuation inversion recovery (FLAIR) image showing subtle hyperintensity involving partial cortex of the right parietal lobe on day 13 after onset; **(C)** FLAIR image demonstrating resolution of the subtle hyperintensity on day 57 after onset; **(D)** Brain MRI with no abnormal signals at 4 months post-onset. Patient 2: **(E, F)** Normal brain MRI on day 2 after onset and 33 months post-onset. Patient 3: **(G, H)** Normal brain MRI on day 2 after onset and 16 months post-onset.

This study had several limitations. First, as a single-center, retrospective case series with a small sample size, potential selection bias may exist. Second, annual cranial MRI surveillance was not conducted throughout the follow-up period; thus, subclinical radiological diseases without overt clinical manifestations could not be ruled out. Therefore, larger-scale prospective studies with standardized serial cranial MRI monitoring during follow-up are warranted to address these limitations and further validate our findings.

## Conclusion

MOG antibody-associated isolated seizures, defined as seizures without encephalopathy, focal neurological deficits, or abnormal cranial MRI findings, represent a distinct and often underrecognized phenotype within the MOGAD spectrum. Although this phenotype lacks specific clinical features, MOG antibody testing is recommended for pediatric patients with the following characteristics: school-age onset, focal motor seizures, elevated ESR and CSF pleocytosis, and no clinical or radiological evidence of encephalitis or demyelination.

Immunotherapy may partially contribute to favorable clinical outcomes in pediatric patients with MOG antibody-associated isolated seizures. However, subsequent clinical or radiological demyelinating events may still occur following this initial phenotype. Due to the limited sample size of the present study, uncertainties persist regarding the risk factors for seizure recurrence and demyelination, as well as the potential of initial immunotherapy to prevent subsequent demyelination. Therefore, further large-sample cohort studies are needed to better characterize the clinical features of this phenotype, identify risk factors for recurrence and demyelination, establish optimal treatment strategies, and assess long-term prognosis.

The study was approved by the Medical Ethics Committee of Hebei Children’s Hospital, affiliated with Hebei Medical University. Informed consent was obtained from the parents or legal guardians of the participating children.

## Data Availability

The raw data supporting the conclusions of this article will be made available by the authors, without undue reservation.
